# Deformation of Composite Laminates Induced by Surface Bonded and Embedded Piezoelectric Actuators

**DOI:** 10.3390/ma13143201

**Published:** 2020-07-17

**Authors:** Shiuh-Chuan Her, Han-Yung Chen

**Affiliations:** Department of Mechanical Engineering, Yuan-Ze University, 135 Yuan-Tong Road, Chung-Li 320, Taiwan; s965025@mail.yzu.edu.tw

**Keywords:** piezoelectric actuator, composite laminate, deflection, deformed shape

## Abstract

In this work, piezoelectric (PZT) actuators were surface bonded on or embedded in a composite laminate and subjected to an electric voltage across the thickness, resulting in a bending effect on the composite laminate. An analytical expression of the deflection of a simply supported cross-ply composite laminate induced by distributed piezoelectric actuators was derived on the basis of classical plate theory and composite mechanics. The theoretical solution can be used to predict the deformation of the composite laminate. Series of parametric studies were performed to investigate the effects of location, size, and embedded depth of PZT actuators on the composite laminate deformation. The analytical predictions were verified with finite element results. A close agreement was achieved. It demonstrated that the present approach provided a simple solution to predict and control the deformed shape of a composite laminate induced by distributed PZT actuators.

## 1. Introduction

Composite materials provide properties that cannot be obtained by their constituents alone. Some of the properties that can be enhanced by forming a composite material are high specific stiffness, light weight, good corrosion and wear resistances, and long fatigue life. These properties make composites become promising materials. The implementation of sensors and actuators to conduct a self-monitoring operation has introduced a new class of structures denoted as “smart structures”. Smart structures incorporating piezoelectric devices to sense and actuate the structure could be utilized in a variety of engineering applications, such as aircraft structures, satellites, large space structures, and so forth [1]. Composite laminates are very suitable to incorporate a network of sensors and actuators to form a smart structure. To advance smart structure further, it is important to be able to predict the deformation induced by the PZT actuator so that the structure can respond to the environmental change appropriately. Thus, a thorough and comprehensive understanding of piezoelectric actuation is necessary.

Smart structures have the ability to adapt environment change through shape or material property modifications. The applications of this technology are numerous, including vibration and buckling control, shape control, damage assessment, and active noise reduction. There are various actuators available for smart structures, such as magnetostrictive actuators [2], electro-rheological actuators [3], shape memory alloy actuators [4], and piezoelectric ceramics actuators. Among them, PZT actuators possess some advantages compared with the other actuators, such as fast response speed, high position accuracy, and not affected by magnetic field [5]. The piezoelectric effect provides the possibility of energy transformation between the mechanical and electrical domain. Electrical voltage exerted on the PZT can be utilized to control the motion of elastic structure, resulting in the PZT actuation on a structure. Mechanical strain applies on the PZT yielding an electrical voltage output which can be used as a sensor. Taking advantage of actuation and sensing capabilities in combination with intelligent electric circuits, vibrational energy might be converted into electrical energy and stored by batteries, leading to energy harvesting [6]. Moreover, external power source is not required for piezoelectric actuator, which results in the availability for compact and low-power consuming products [7]. PZT actuators have been widely used in a variety of engineering applications, such as damage identification, structure health monitoring, energy harvesting, and vibration control [8]. PZT has been considered as low-cost, lightweight, and easy-to-implement material and provides the sensing and actuation capabilities for passive and active vibration control [9]. It can be surface bonded to the structure with minimal modification of the original structure or embedded in composite laminate structures. Based on the working principles, PZT actuators are characterized into direct driving type [10], ultrasonic type [11], inchworm type [12], and inertial types [13]. Sharma et al. [14] proposed a Lead Zirconate-Titanate (PZT)/poly-dimethylsiloxane (PDMS)-based flexible composite and investigated its application in the active damping of vibration control. Shape control involves manipulating structure’s shape to an appropriate shape in response to the environmental change by integrated actuators. The aim of shape control is to achieve the shape as close as possible to the desired shape by determining the input parameters. Among the various parameters, actuator size, location, and electrical voltage are the most dominate factors for the shape control. Song et al. [15] theoretically and experimentally investigated the active shape control of an antenna reflector using piezoceramic actuators attached on ribs. For a given desired shape, they proposed a closed-loop iterative shape method to obtain high-precision shape control. Austin et al. [16] designed and constructed adaptive wings utilizing PZT actuators to modify the desired airfoil shape to minimize and improve the aerodynamic performance. Sabatini et al. [17] studied the active vibration control of a flexible space manipulator during on orbit operation based on direct velocity feedback via PZT actuator. The number of PZT actuators, their placement, and their operational mode were presented to obtain maximum performance in terms of elastic oscillations reduction and power consumption. Azadi et al. [18] proposed a mathematical model based on the Lagrange–Rayleigh–Ritz technique to achieve the satellite’s large angle trajectory tracking and suppress the vibration of the appendages using PZT actuators. Wang et al. [19] presented an integrated design of a composite laminate plate with surface bonded piezocomposite actuators to maximize static bending and twisting deformation of the plate under applied voltages for the actuators. The design variables include thickness, stacking sequences, ply angles of the substrate laminated plate, and locations and orientations of PZT actuators. Irschik and Nader [20] used Mohr’s analogy for determining the proper location and the length of a piezoelectric patch to control the deformation and the cross-sectional rotation of a beam. Schoeftner et al. [6] proposed a theoretical model for static and dynamic shape control of a beam based on one-dimensional extended Bernoulli–Euler theory, which was verified by three-dimensional finite element calculations with ANSYS. Zhang et al. [21] developed nonlinear finite element (FE) models using various nonlinear shell theories based on the first-order shear deformation (FOSD) hypothesis for shape and vibration control of structures undergoing large displacements and rotations. Their results show that, by applying an appropriate voltage, a desired shape can be achieved, as well as the vibration can be significantly suppressed. Nguyen and Tong [22] proposed a finite element model for analyzing static deformation of a smart composite plate with non-rectangular PZT actuators. The mechanical deformation of the smart composite plate was modeled using third-order plate theory, while the electric field was simulated based on a layer-wise theory. Ren [23] reported a theoretical model based on Rayleigh–Ritz principle to investigate the cured shape and deformation control of thin arbitrary lay-up composite laminates using PZT actuators. Gohari et al. [24] presented an explicit analytical solution based on the linear piezoelectricity and plate theories for obtaining twisting deformation and optimal shape control of smart laminated cantilever composite plates/beams using inclined piezoelectric actuators. The effect of various parameters including electro-mechanical twisting coupling, layup thickness, actuators size, placement, and inclination angle were investigated. Chee et al. [25] developed a mathematical model based on a higher-order displacement for the determination of the PZT actuator orientation in the application to the shape control of a composite plate. The performance of shape control was examined via the displacement response of the composite plate. Lin and Nien [26] developed a finite element model to study the dynamic and static responses of composite laminated plates containing distributed piezoceramic actuators under both mechanical and electrical loadings. The effectiveness of using PZT actuators to tune the deformation and control the shape of composite laminated plates was presented.

The present study investigated the actuation capability of PZT actuator. The aim of this study was to develop a theoretical model to predict the deformation of the composite laminate plate induced by PZT actuators and illustrate how the shape control of a composite laminate plate can be achieved via PZT actuators. PZT actuators were surface bonded onto or embedded into a cross-ply composite laminate plate and subjected to an electrical voltage, resulting in a bending effect on the composite laminate. The analytical expression of the deflection of a simply supported composite laminate was derived on the basis of composite mechanics and plate theory. Series of parametric studies were performed to investigate the effects of location, size, and embedded depth of PZT actuators on the composite laminate deformation. An example is presented to illustrate that the deformed shape of the composite laminate can be effectively activated by distributed PZT actuators.

## 2. Bending Moments Induced by PZT Actuator

For an unconstrained piezoelectric actuator, when it is activated by an electric voltage along its polarization direction (*z*-direction), equal strains in both *x* and *y* directions can be induced. The magnitude of the strains in both *x* and *y* directions is a function of applied voltage V, PZT thickness $t_{pe}$, and the piezoelectric-strain constant $d_{31}$ as follow

(1) $${\left(\varepsilon_{pe}\right)}_{x}={\left(\varepsilon_{pe}\right)}_{y}=\frac{d_{31}}{t_{pe}}V$$

**Figure 1:**
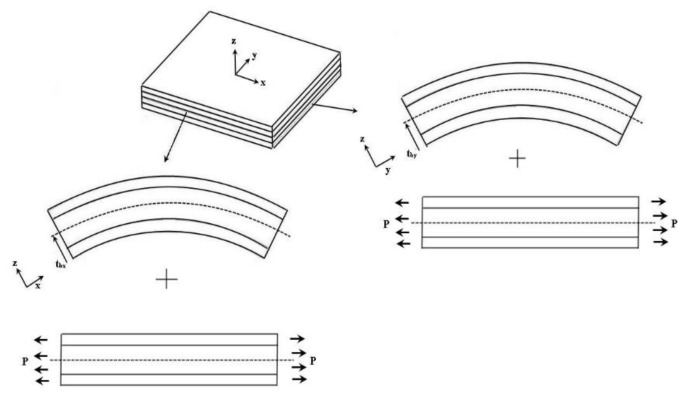
Strain in composite laminate decomposed into uniform and bending components.

In this work, PZT actuator can be either surface bonded on or embedded in a cross-ply composite laminate plate. The strain of the composite laminate induced by the PZT actuator can be decomposed into two components, uniform component and bending component, respectively, as shown in Figure 1.

The normal strains along the x and y directions in the ith layer of the composite laminate are expressed as

(2) $$\varepsilon_{x}^{\left(i\right)}=c_{x}+\frac{z-t_{\mathrm{bx}}}{r_{x}}$$

(3) $$\varepsilon_{y}^{\left(i\right)}=c_{y}+\frac{z-t_{\mathrm{by}}}{r_{y}}$$

where $c_{x}$ and $c_{y}$ are the uniform strain components along the x and y directions, respectively; $r_{x}$ and $r_{y}$ are the radii of curvature in the x-z and y-z planes, respectively; $t_{\mathrm{bx}}$ and $t_{\mathrm{by}}$ are the locations of bending axes in the x-*z* and y-z planes, respectively; and *z* is the coordinate along the thickness direction.

The normal stresses along the x and y directions in the ith layer of the composite laminate are written as

(4) $$\sigma_{x}^{\left(i\right)}=\frac{E_{x}^{\left(i\right)}}{1-\nu_{12}^{\left(i\right)}\nu_{21}^{\left(i\right)}}\left(\varepsilon_{x}^{\left(i\right)}-\frac{d_{31}^{\left(i\right)}}{t_{i}}V\right)+\frac{\nu_{12}^{\left(i\right)}E_{y}^{\left(i\right)}}{1-\nu_{12}^{\left(i\right)}\nu_{21}^{\left(i\right)}}\left(\varepsilon_{y}^{\left(i\right)}-\frac{d_{31}^{\left(i\right)}}{t_{i}}V\right)$$

(5) $$\sigma_{y}^{\left(i\right)}=\frac{\nu_{12}^{\left(i\right)}E_{y}^{\left(i\right)}}{1-\nu_{12}^{\left(i\right)}\nu_{21}^{\left(i\right)}}\left(\varepsilon_{x}^{\left(i\right)}-\frac{d_{31}^{\left(i\right)}}{t_{i}}V\right)+\frac{E_{y}^{\left(i\right)}}{1-\nu_{12}^{\left(i\right)}\nu_{21}^{\left(i\right)}}\left(\varepsilon_{y}^{\left(i\right)}-\frac{d_{31}^{\left(i\right)}}{t_{i}}V\right)$$

where $E_{x}^{\left(i\right)}$ and $E_{y}^{\left(i\right)}$ are the Young’s moduli along the x and y directions of the ith layer, respectively; $\nu_{12}^{\left(i\right)}$ and $\nu_{21}^{\left(i\right)}$ are Poisson’s ratios of the ith layer; and $t_{i}$ and $d_{31}^{\left(i\right)}$ are the thickness and piezoelectric-strain constant of the ith layer, respectively. It should be noted that the ith layer can be either a composite layer or PZT layer. If the ith layer is a composite layer, $d_{31}^{\left(i\right)}=0$.

Substituting Equations (2) and (3) into Equations (4) and (5) leads to

(6) $$\sigma_{x}^{\left(i\right)}=\frac{E_{x}^{\left(i\right)}}{1-\nu_{12}^{\left(i\right)}\nu_{21}^{\left(i\right)}}\left(c_{x}+\frac{z-t_{\mathrm{bx}}}{r_{x}}-\frac{d_{31}^{\left(i\right)}}{t_{i}}V\right)+\frac{\nu_{12}^{\left(i\right)}E_{y}^{\left(i\right)}}{1-\nu_{12}^{\left(i\right)}\nu_{21}^{\left(i\right)}}\left(c_{y}+\frac{z-t_{\mathrm{by}}}{r_{y}}-\frac{d_{32}^{\left(i\right)}}{t_{i}}V\right)$$

(7) $$\sigma_{y}^{\left(i\right)}=\frac{\nu_{12}^{\left(i\right)}E_{y}^{\left(i\right)}}{1-\nu_{12}^{\left(i\right)}\nu_{21}^{\left(i\right)}}\left(c_{x}+\frac{z-t_{\mathrm{bx}}}{r_{x}}-\frac{d_{31}^{\left(i\right)}}{t_{i}}V\right)+\frac{E_{y}^{\left(i\right)}}{1-\nu_{12}^{\left(i\right)}\nu_{21}^{\left(i\right)}}\left(c_{y}+\frac{z-t_{\mathrm{by}}}{r_{y}}-\frac{d_{32}^{\left(i\right)}}{t_{i}}V\right)$$

There are six unknown constants, $c_{x}{\;,\;c}_{y}{\;,\;r}_{x}{\;,\;r}_{y}{\;,\;t}_{\mathrm{bx}}{\;\mathrm{and}\;t}_{\mathrm{by}}$, to be determined by the following conditions.

(1) The resultant forces due to the uniform tension along the *x* and *y* direction are equal to zero.

(8) $$\overset{n}{\underset{i=1}{\sum }}E_{x}^{\left(i\right)}\left(c_{x}-\frac{d_{31}^{\left(i\right)}}{t_{i}}V\right)t_{i}=0$$

(9) $$\sum_{i=1}^{n}E_{y}^{\left(i\right)}\left(c_{y}-\frac{d_{31}^{\left(i\right)}}{t_{i}}V\right)t_{i}=0$$

The constants of $c_{x}{\;\mathrm{and}\;c}_{y}$ can be solved by substituting the Young’s modulus in *x*-direction $E_{x}^{\left(i\right)}$ and Young’s modulus in *y*-direction$\;E_{y}^{\left(i\right)}$, layer thickness $t_{i}$, piezoelectric-strain constant $d_{31}^{\left(i\right)}$ of each layer, and electrical voltage V into Equations (8) and (9).

(10) $$c_{x}=\frac{\sum_{i=1}^{n}E_{x}^{\left(i\right)}d_{31}^{\left(i\right)}V}{\sum_{i=1}^{n}E_{x}^{\left(i\right)}t_{i}}$$

(11) $$c_{y}=\frac{\sum_{i=1}^{n}E_{y}^{\left(i\right)}d_{32}^{\left(i\right)}V}{\sum_{i=1}^{n}E_{y}^{\left(i\right)}t_{i}}$$

(2) The resultant forces due to the bending along the *x* and *y* direction are equal to zero:

(12) $$\overset{n}{\underset{i=1}{\sum }}\int_{z_{i-1}}^{z_{i}}\frac{E_{x}^{\left(i\right)}\left(z-t_{\mathrm{bx}}\right)}{r_{x}}\mathrm{dz}=0$$

(13) $$\overset{n}{\underset{i=1}{\sum }}\int_{z_{i-1}}^{z_{i}}\frac{E_{y}^{\left(i\right)}\left(z-t_{\mathrm{by}}\right)}{r_{y}}dz=0$$

where $z_{i}{\;\mathrm{and}\;z}_{i-1}$ are the *z* coordinates at the top and bottom surfaces of the ith layer, respectively. The thickness of the ith layer $t_{i}{=z}_{i}{-z}_{i-1}$; $z_{0}=0$.

Multiplying Equations (12) and (13) by $r_{x}$ and $r_{y}$, respectively, leads to the determination of the constants of $t_{\mathrm{bx}}{\;\mathrm{and}\;t}_{\mathrm{by}}$ as follow.

(14) $$t_{\mathrm{bx}}=\frac{\sum_{i=1}^{n}E_{x}^{\left(i\right)}\left(z_{i}^{2}-z_{i-1}^{2}\right)}{2\sum_{i=1}^{n}E_{x}^{\left(i\right)}t_{i}}=\frac{\sum_{i=1}^{n}E_{x}^{\left(i\right)}t_{i}\left(2{\;z}_{i-1}-t_{i}\right)}{2\sum_{i=1}^{n}E_{x}^{\left(i\right)}t_{i}}$$

(15) $$t_{\mathrm{by}}=\frac{\sum_{i=1}^{n}E_{y}^{\left(i\right)}\left(z_{i}^{2}-z_{i-1}^{2}\right)}{2\sum_{i=1}^{n}E_{y}^{\left(i\right)}t_{i}}=\frac{\sum_{i=1}^{n}E_{y}^{\left(i\right)}t_{i}\left(2{\;z}_{i-1}-t_{i}\right)}{2\sum_{i=1}^{n}E_{y}^{\left(i\right)}t_{i}}$$

(3) The resultant bending moments in the x and y directions are equal to zero.

(16) $$\sum_{i=1}^{n}\int_{z_{i-1}}^{z_{i}}\sigma_{x}^{\left(i\right)}\left(z-t_{\mathrm{bx}}\right)dz=0$$

(17) $$\overset{n}{\underset{i=1}{\sum }}\int_{z_{i-1}}^{z_{i}}\sigma_{y}^{\left(i\right)}\left(z-t_{\mathrm{by}}\right)dz=0$$

substituting Equations (10), (11), (14) and (15) into Equations (6) and (7), and then substituting into Equations (16) and (17) leads to

(18) $$\begin{array}{l} \sum_{i=1}^{n}\int_{z_{i-1}}^{z_{i}}\left\lceil \frac{E_{x}^{\left(i\right)}}{1-\nu_{12}^{\left(i\right)}\nu_{21}^{\left(i\right)}}\left(c_{x}+\frac{z-t_{\mathrm{bx}}}{r_{x}}-\frac{d_{31}^{\left(i\right)}}{t_{i}}V\right)+\frac{\nu_{12}^{\left(i\right)}E_{y}^{\left(i\right)}}{1-\nu_{12}^{\left(i\right)}\nu_{21}^{\left(i\right)}}\left(c_{y}+\frac{z-t_{\mathrm{by}}}{r_{y}}-\frac{d_{32}^{\left(i\right)}}{t_{i}}V\right)\right\rceil \\ \left(z-\frac{\sum_{i=1}^{n}E_{x}^{\left(i\right)}t_{i}\left(2z_{i-1}-t_{i}\right)}{2\sum_{i=1}^{n}E_{x}^{\left(i\right)}t_{i}}\right)dz=0 \end{array}$$

(19) $$\begin{array}{l} \sum_{i=1}^{n}\int_{z_{i-1}}^{z_{i}}\left\lceil \frac{\nu_{12}^{\left(i\right)}E_{y}^{\left(i\right)}}{1-\nu_{12}^{\left(i\right)}\nu_{21}^{\left(i\right)}}\left(c_{x}+\frac{z-t_{\mathrm{bx}}}{r_{x}}-\frac{d_{31}^{\left(i\right)}}{t_{i}}V\right)+\frac{E_{y}^{\left(i\right)}}{1-\nu_{12}^{\left(i\right)}\nu_{21}^{\left(i\right)}}\left(c_{y}+\frac{z-t_{\mathrm{by}}}{r_{y}}-\frac{d_{32}^{\left(i\right)}}{t_{i}}V\right)\right\rceil \\ \left(z-\frac{\sum_{i=1}^{n}E_{y}^{\left(i\right)}t_{i}\left(2z_{i-1}-t_{i}\right)}{2\sum_{i=1}^{n}E_{y}^{\left(i\right)}t_{i}}\right)dz=0 \end{array}$$

The constants of $r_{x}{\;\mathrm{and}\;r}_{y}$ are readily determined by substituting $c_{x}$ and $c_{y}$ from Equations (10) and (11) and $t_{\mathrm{bx}}$ and $t_{\mathrm{by}}$ from Equations (14) and (15) into Equations (18) and (19).

**Figure 2:**
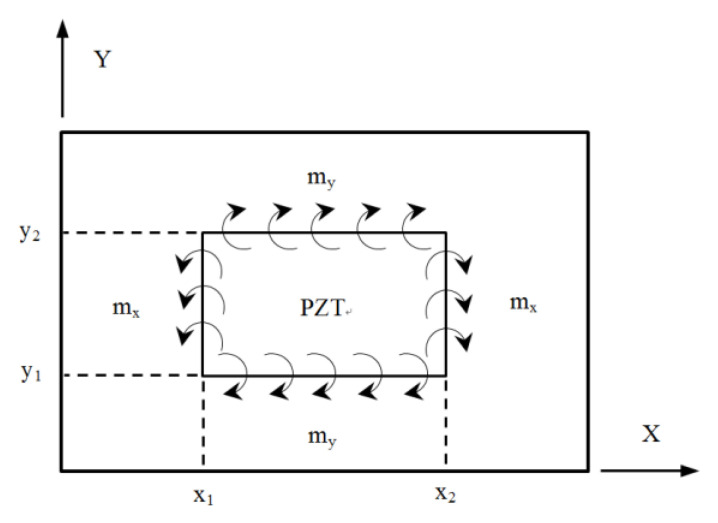
Bending moment exerted on the composite laminate by PZT actuator.

Bending moments exerted on the composite laminate by the PZT actuator, as shown in Figure 2, can be written as follows.

(20) $$m_{x}=\int_{z_{a}}^{z_{b}}\sigma_{x}^{\mathrm{Pe}}\left(z-t_{\mathrm{bx}}\right)dz=\int_{z_{a}}^{z_{b}}E_{x}^{\mathrm{Pe}}{\left(\frac{z-t_{\mathrm{bx}}}{r_{x}}\right)}^{2}dz$$

(21) $$m_{y}=\int_{z_{a}}^{z_{b}}\sigma_{y}^{\mathrm{Pe}}\left(z-t_{\mathrm{by}}\right)dz=\int_{z_{a}}^{z_{b}}E_{y}^{\mathrm{Pe}}{\left(\frac{z-t_{\mathrm{by}}}{r_{y}}\right)}^{2}dz$$

where $E_{x}^{\mathrm{pe}}{\;\mathrm{and}\;E}_{y}^{\mathrm{pe}}$ are the Young’s moduli of the PZT actuator in the x and y directions, respectively. $z_{b}{\;\mathrm{and}\;z}_{a}$ are the *z* coordinates at the top and bottom surfaces of PZT actuator in the *z* direction, respectively.

## 3. Deflection of A Simply Supported Cross Ply Composite Laminate Plate

Deflection of the composite laminate plate is considered as the most appropriate variable to describe the deformation. Thus, the present approach focuses on the derivation of the displacement of the composite laminate. Utilizing the classical plate theory, the governing differential equation of the plate can be expressed in terms of the internal bending moments ($M_{x}{\;,\;M}_{y}{\;,\;M}_{\mathrm{xy}})\;$and external bending moments ($m_{x}\;,\;\;m_{y})\;$exerted by the PZT actuator as follow.

(22) $$\frac{\partial^{2}(M_{x}-m_{x})}{\partial x^{2}}+2\frac{\partial^{2}M_{xy}}{\partial x\partial y}+\frac{\partial^{2}(M_{y}-m_{y})}{\partial y^{2}}=0$$

The external bending moments induced by PZT actuator, which is surface bonded or embedded in the composite laminate, as shown in Figure 2, can be incorporated with unit step functions and rewritten as

(23) $$m_{x}=\left[h\left(x-x_{1}\right)-h\left(x-x_{2}\right)\right]\left[h\left(y-y_{1}\right)-h\left(y-y_{2}\right)\right]\int_{z_{a}}^{z_{b}}E_{x}^{\mathrm{Pe}}{\left(\frac{z-t_{\mathrm{bx}}}{r_{x}}\right)}^{2}\mathrm{dz}$$

(24) $$m_{y}=\left[h\left(x-x_{1}\right)-h\left(x-x_{2}\right)\right]\left[h\left(y-y_{1}\right)-h\left(y-y_{2}\right)\right]\int_{z_{a}}^{z_{b}}E_{y}^{\mathrm{Pe}}{\left(\frac{z-t_{\mathrm{by}}}{r_{y}}\right)}^{2}\mathrm{dz}$$

The internal bending moments in the cross-ply composite laminate plate are expressed as follow.

(25) $$\left\{\begin{array}{c} M_{x}\\ M_{y}\\ M_{xy} \end{array}\right\}=\left[\begin{array}{ccc} D_{11} & D_{12} & D_{16}\\ D_{12} & D_{22} & D_{26}\\ D_{16} & D_{26} & D_{66} \end{array}\right]\left\{\begin{array}{c} \kappa_{x}\\ \kappa_{y}\\ \kappa_{xy} \end{array}\right\}\;{\left[D\right]}_{3\times 3}=\frac{1}{3}\sum_{i=1}^{N}\left[\bar{Q}\right]{\;}_{3\times 3}^{i}\left(Z_{i}^{3}-Z_{i-1}^{3}\right)$$

where i and N denote the ith layer and number of layers in the composite laminate, respectively; ${\left[\bar{Q}\right]}_{3\times 3}^{i}$ represent the stiffness matrix of the ith layer; $z_{i}\;\mathrm{and}\;z_{i-1}\;$are the *z* coordinates of the top and bottom surfaces of the ith layer along the thickness direction, respectively; and $D_{16}=D_{26}=0$ for the cross-ply composite laminate.

$\kappa_{x}\;,\;k_{y},\;\mathrm{and}\;k_{\mathrm{xy}}$ represent the curvatures of the composite laminate, which can be written in terms of the deflection as follow.

(26) $$\kappa_{x}=-\frac{\partial^{2}W}{\partial x^{2}}$$

(27) $$\kappa_{y}=-\frac{\partial^{2}W}{\partial y^{2}}$$

(28) $$\kappa_{\mathrm{xy}}=-2\frac{\partial^{2}W}{\partial x\partial y}$$

Substituting Equations (23)–(28) into Equation (22), leads to

(29) $$D_{11}\frac{\partial^{4}W}{\partial x^{4}}+2H_{1}\frac{\partial^{4}W}{\partial x^{2}\partial y^{2}}+D_{22}\frac{\partial^{4}W}{\partial y^{4}}=\frac{\partial^{2}m_{x}}{\partial x^{2}}+\frac{\partial^{2}m_{y}}{\partial y^{2}}=P\left(x,y\right)\;H_{1}=D_{12}+2D_{66}$$

(30) $$p\left(x,y\right)=\left[\delta^{\prime }\left(x-x_{1}\right)-\;\delta^{\prime }\left(x-x_{2}\right)\right]\left[h\left(y-y_{1}\right)-h\left(y-y_{2}\right)\right]\int_{z_{a}}^{z_{b}}E_{x}^{\mathrm{Pe}}{\left(\frac{z-t_{\mathrm{bx}}}{r_{x}}\right)}^{2}\mathrm{dz}\;+\left[h\left(x-x_{1}\right)-h\left(x-x_{2}\right)\right]\left[\delta^{\prime }\left(y-y_{1}\right)-\;\delta^{\prime }\left(y-y_{2}\right)\right]\int_{z_{a}}^{z_{b}}E_{y}^{\mathrm{Pe}}{\left(\frac{z-t_{\mathrm{by}}}{r_{y}}\right)}^{2}\mathrm{dz}$$

For a simply supported plate, the deflection can be expressed in terms of Fourier series as follow.

(31) $$W\left(x,y\right)=\overset{\infty }{\underset{m=1}{\sum }}\overset{\infty }{\underset{n=1}{\sum }}W_{\mathrm{mn}}\sin\frac{m\pi x}{a}\sin\frac{n\pi y}{b}$$

where a and b are the length and width of the composite laminate plate, respectively. $W_{\mathrm{mn}}$ is the undeterminant constant.

The forcing function P(*x,y*) shown in Equation (30) can be rewritten in terms of Fourier series as follow.

(32) $$P(x,y)=\sum_{m=1}^{\infty }\sum_{n=1}^{\infty }P_{mn}\sin\frac{m\pi x}{a}\sin\frac{n\pi y}{b}\;P_{mn}=\frac{4}{a\times b}\int_{0}^{b}\int_{0}^{a}P\left(x.y\right)sin\frac{m\pi x}{a}sin\frac{n\pi y}{b}dxdy\;=\frac{4}{a\times b}\left[-\frac{m_{y}\gamma_{m}^{2}+m_{x}\gamma_{n}^{2}}{\gamma_{m}\gamma_{n}}\right]\left(cos\gamma_{m}x_{1}-cos\gamma_{m}x_{2}\right)\left(cos\gamma_{n}y_{1}-cos\gamma_{n}y_{2}\right)\;\gamma_{m}=\frac{m\pi }{a}\;\;\gamma_{n}=\frac{n\pi }{b}$$

Substituting Equations (18) and (19) into Equation (16) yields

(33) $$\begin{array}{l} \sum_{m=1}^{\infty }\sum_{n=1}^{\infty }\left\{W_{mn}\left(D_{11}\frac{m^{4}\pi^{4}}{a^{4}}+2H_{1}\frac{m^{2}\pi^{2}}{a^{2}}\frac{n^{2}\pi^{2}}{b^{2}}+D_{22}\frac{n^{4}\pi^{4}}{b^{4}}\right)\right\}\sin\frac{m\pi x}{a}\sin\frac{n\pi y}{b}\\ =\sum_{m=1}^{\infty }\sum_{n=1}^{\infty }P_{mn}\sin\frac{m\pi x}{a}\sin\frac{n\pi y}{b} \end{array}$$

Thus, the constant $W_{\mathrm{mn}}$ is readily to be determined as follow, which leads to the determination of the deflection of the composite laminate plate induced by the PZT actuator.

(34) $$W_{mn}=\frac{P_{mn}}{\left(\frac{m^{4}\pi^{4}}{a^{4}}D_{11}+\frac{m^{2}\pi^{2}}{a^{2}}\frac{n^{2}\pi^{2}}{b^{2}}2H_{1}+\frac{n^{4}\pi^{4}}{b^{4}}D_{22}\right)}$$

## 4. Parametric Studies and Verification

There are several variables such as PZT size, location, embedded depth, orientation, electrical voltage, layup thickness, and stacking sequence which can affect the PZT actuation [24]. Among them, PZT size and location are considered as the most important factors related to the deformation magnitude and deformed shape, respectively. Series of parametric studies were performed to evaluate the influences of the size, location, and embedded depth of PZT actuators on the deformation of the composite laminate plate. Finite element method is a well-developed numerical technique in structural analysis. Due to the lack of experimental equipment, we were not able to validate the present approach with the experimental results. Thus, finite element method was the alternate choice to verify present approach. A numerical example with distributed PZT actuators is presented to illustrate the capability of shape control using PZT actuators. The composite laminate used in this work was Boron/Al with stacking sequence of [0/90/90/0]. The material properties of Boron/Al are presented in Table 1 [27]. The dimensions of the composite laminate plate are length *a* = 500 mm, width *b* = 500 mm, and thickness *t* = 5 mm. PZT-4 was used for the piezoelectric actuator with material properties [28] of elastic modulus *E*_pe_ = 78 GPa, Poisson’s ratio *v*_pe_ *=* 0.31, piezoelectric constant *d_31_* = −1.22 × 10^−10^ V/m, and thickness *t*_pe_ = 0.5 mm. The electric voltage applied on the PZT actuator was −100 V.

### 4.1. The Effect of PZT Size

**Figure 3:**
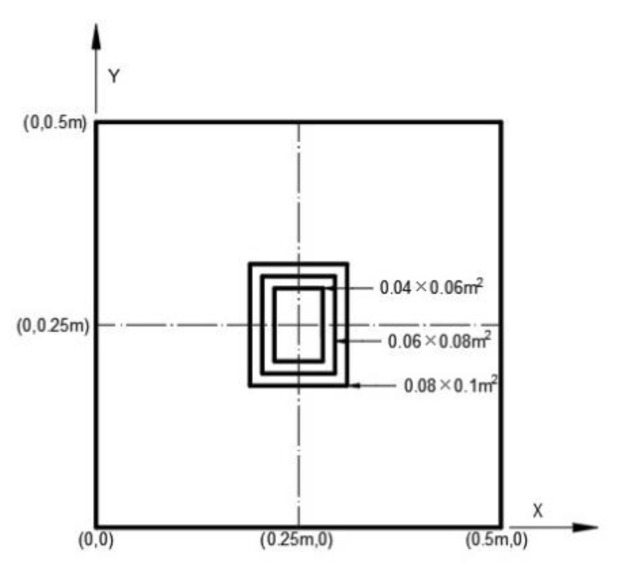
Three different sizes of PZT actuators surface bonded on the center of the composite laminate plate.

**Figure 4:**
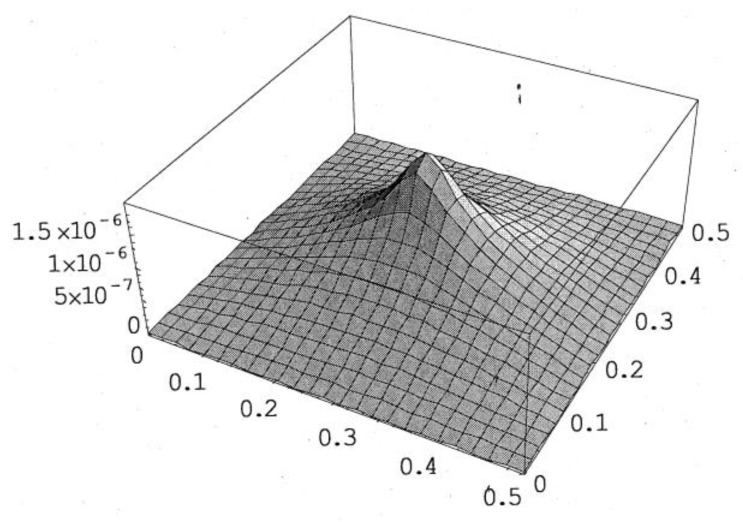
Deformed shape of the composite laminate plate induced by PZT actuator with dimensions of 40 mm × 60 mm × 0.5 mm.

**Figure 5:**
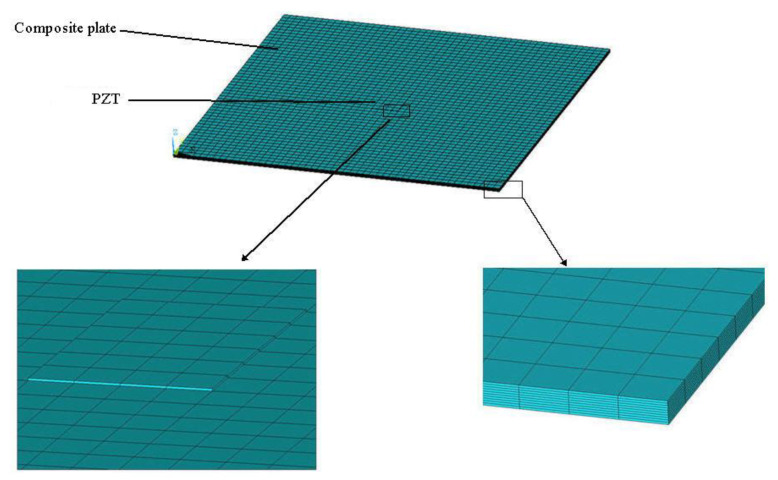
Finite element mesh.

**Figure 6:**
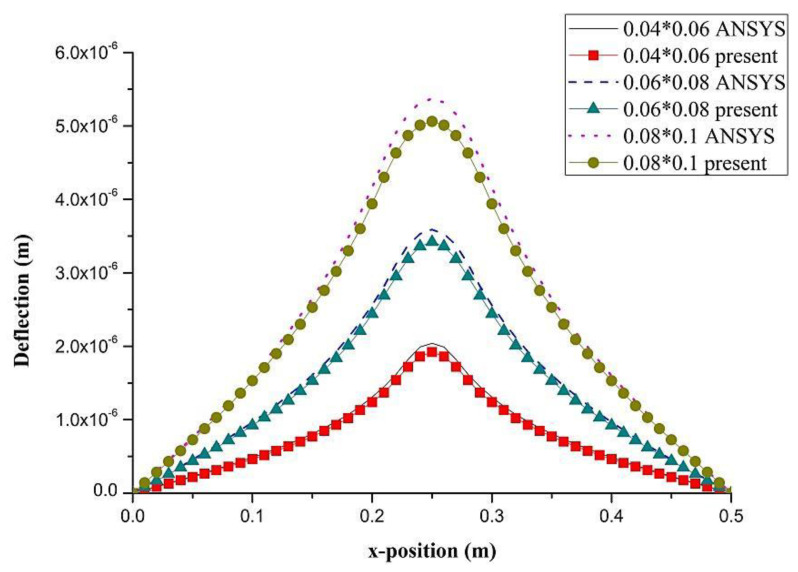
Deflection along the horizontal line (y = 0.25 m) of the composite laminate plate induced by PZT actuator with three different sizes.

**Figure 7:**
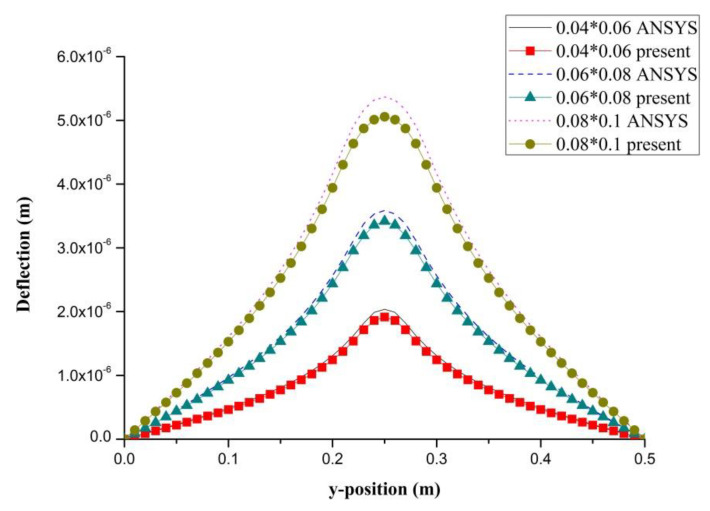
Deflection along the vertical line (x = 0.25 m) of the composite laminate plate induced by a PZT actuator with three different sizes.

PZT actuator was surfaced bonded on the central area of a composite laminate plate with three different sizes of 40 × 60 × 0.5 ${\mathrm{mm}}^{3}$, 60 × 80 × 0.5 ${\mathrm{mm}}^{3}$, and 80 × 100 × 0.5 ${\mathrm{mm}}^{3}$, as shown in Figure 3. The bending moments exerted on the composite laminate induced by the PZT actuator were calculated using Equations (20) and (21), resulting in $m_{x}=3.42\;N\;m/m$ and $m_{y}=3.19\;N\;m/m$. After substituting the bending moments into Equation (32), the deflection of the composite laminate plate was readily determined using Equations (34) and (31). Figure 4 plots the deformation of the composite laminate plate induced by a PZT actuator with dimensions of 40 × 60 × 0.5 mm^3^. Finite element software ANSYS 2020 (ANSYS Inc., Canonsburg, PA, USA) was employed to verify the present approach. In the finite element analysis, SOLID 5 and SOLID 45 elements were adopted for the PZT actuator and composite laminate, respectively. Figure 5 illustrates the 3D finite element mesh. The load exerted on the PZT actuator was a voltage employed between the upper and lower surfaces of the SOLID 5 elements, leading to an electric field along the poling direction of the actuator. Figure 6 and Figure 7 plot the deflections of the composite laminate plate induced by a PZT actuator with three different sizes along the horizontal line y = 0.25 m and vertical line x = 0.25 m, respectively. It can be observed that the deformation of the composite laminate plate predicted by the present approach agrees well with the finite element results. The deflection is increasing with the increase of the PZT size.

### 4.2. The Effect of PZT Location

**Figure 8:**
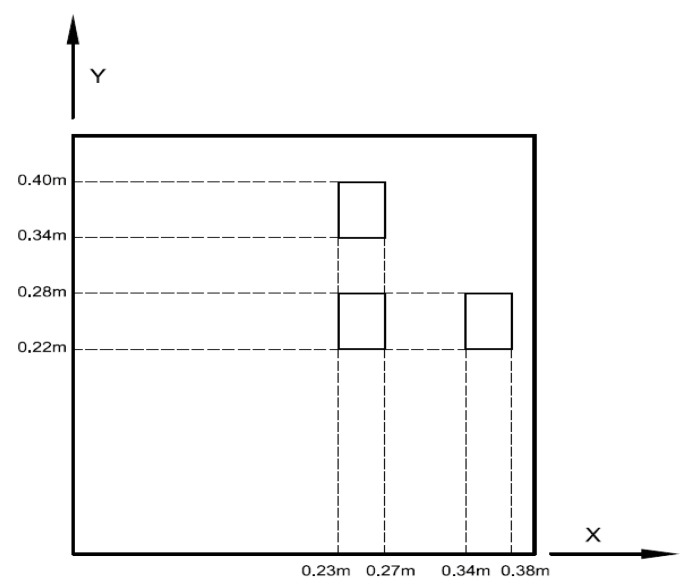
PZT actuator surface bonded on the composite laminate plate at three different locations.

**Figure 9:**
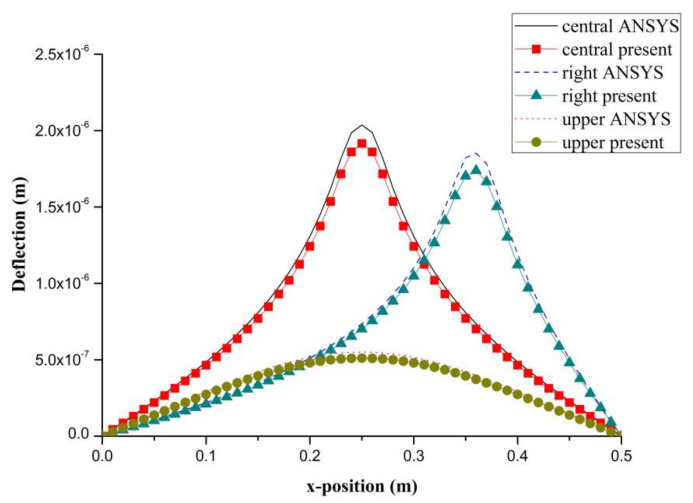
Deflection along the horizontal line (y = 0.25 m) of the composite laminate plate induced by a PZT actuator at three different locations.

**Figure 10:**
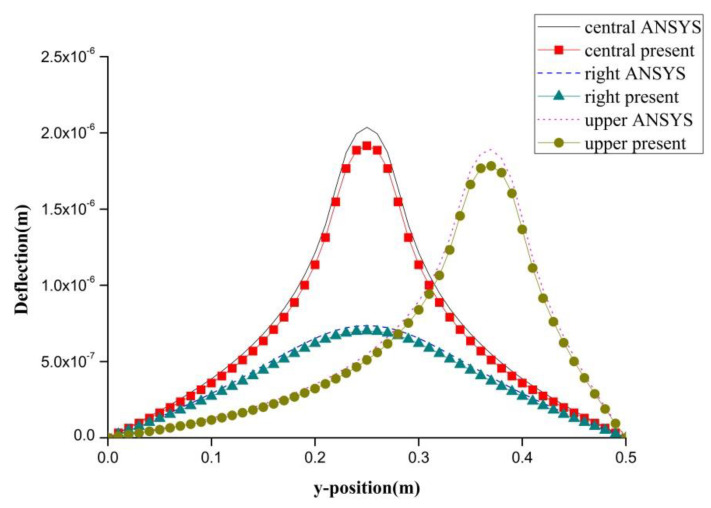
Deflection along the vertical line (*x* = 0.25 m) of the composite laminate plate induced by a PZT actuator at three different locations.

**Figure 11:**
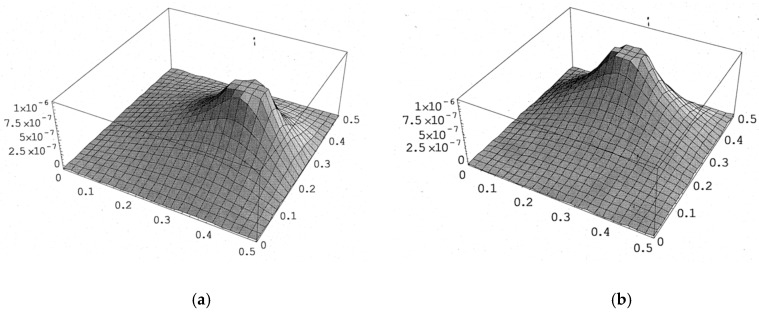
The deformed shape of the composite laminate plate with PZT actuator located at the right and top positions of the plate. (**a**) PZT actuator located at right of composite plate. (**b**) PZT actuator located at top of composite plate.

PZT actuator was surface bonded on the composite laminate at three different locations (central, right, and upper locations), as shown in Figure 8. The dimensions of the PZT actuator were 40 mm × 60 mm × 0.5 mm. The deflections of the composite laminate plate along the horizontal line *y* = 0.25 m and vertical line *x* = 0.25 m induced by a PZT actuator placed at three different locations are plotted in Figure 9 and Figure 10, respectively. It appears that a large deformation of the composite laminate plate is occurred at the location where the PZT actuator is bonded. This demonstrates that PZT actuators can effectively activate the deformation of a composite laminate plate at the desired location. Three-dimensional deformed shapes are plotted in Figure 11. It can be observed that the deformed shape is significantly affected by the placement of the PZT actuator.

### 4.3. The effect of PZT Embedded Depth

**Figure 12:**
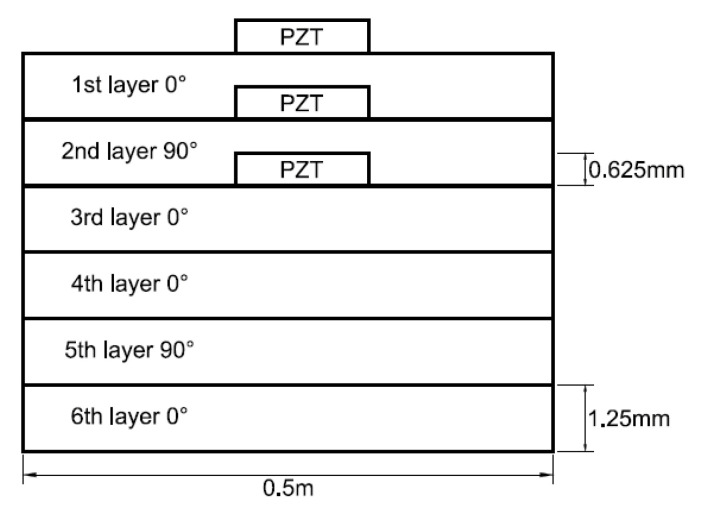
PZT actuators surface bonded, embedded in the first and second layers of the composite laminate.

**Figure 13:**
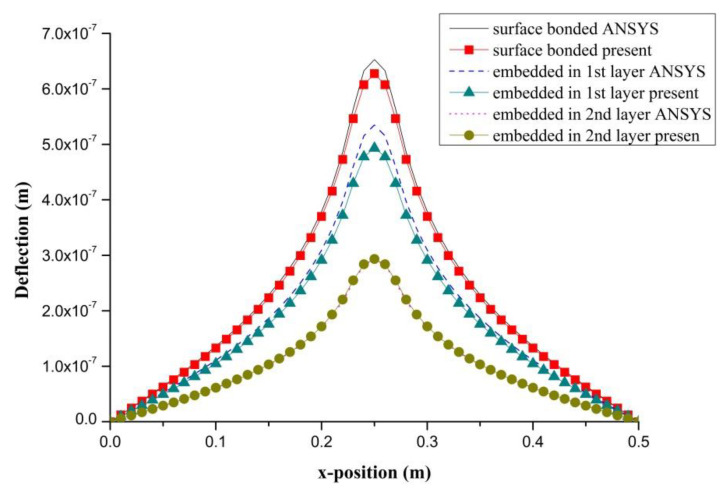
Deflection along the horizontal line (*y* = 0.25 m) of the composite laminate plate induced by a PZT actuator embedded in different depths.

**Figure 14:**
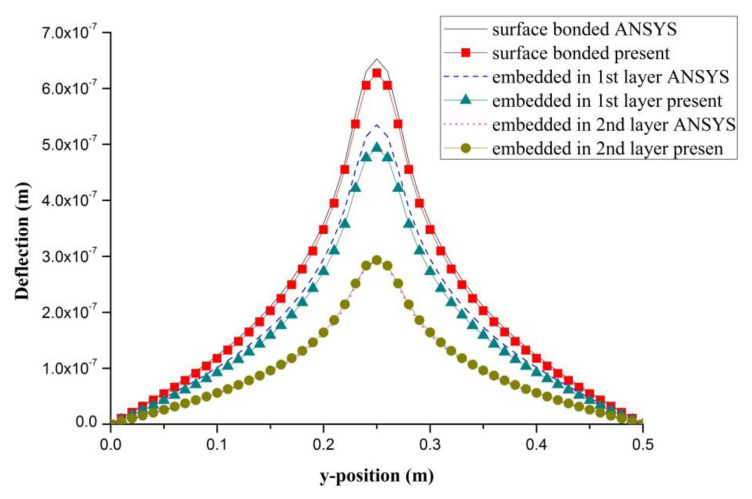
Deflection along the vertical line (*x* = 0.25 m) of the composite laminate plate induced by a PZT actuator embedded in different depths.

PZT actuator was embedded in a composite laminate with stacking sequence of [0/90/0${]}_{s}$. The dimensions of the PZT actuator and Boron/Al layer were 40 × 40 × 0.625 mm^3^ and 500 × 500 × 1.25 mm^3^, respectively. PZT actuators were embedded in the first and second layers located at the center of the composite laminate, as shown in Figure 12. In addition, PZT actuator surface bonded on the composite laminate was also included as reference. The deflections of the composite laminate plate along the horizontal line (*y* = 0.25 m) and vertical line (*x* = 0.25 m) induced by the embedded PZT actuator are presented in Figure 13 and Figure 14, respectively. It can be observed that the deflection of the composite laminate is decreasing with the increase of the PZT embedded depth. The deformation induced by the surface bonded PZT actuator is larger than that of embedded PZT actuator. A close agreement between the present approach and finite element result demonstrates that the proposed approach can be used to predict the deformation of a composite laminate plate induced by a series of distributed PZT actuators.

### 4.4. Distributed PZT Actuators

**Figure 15:**
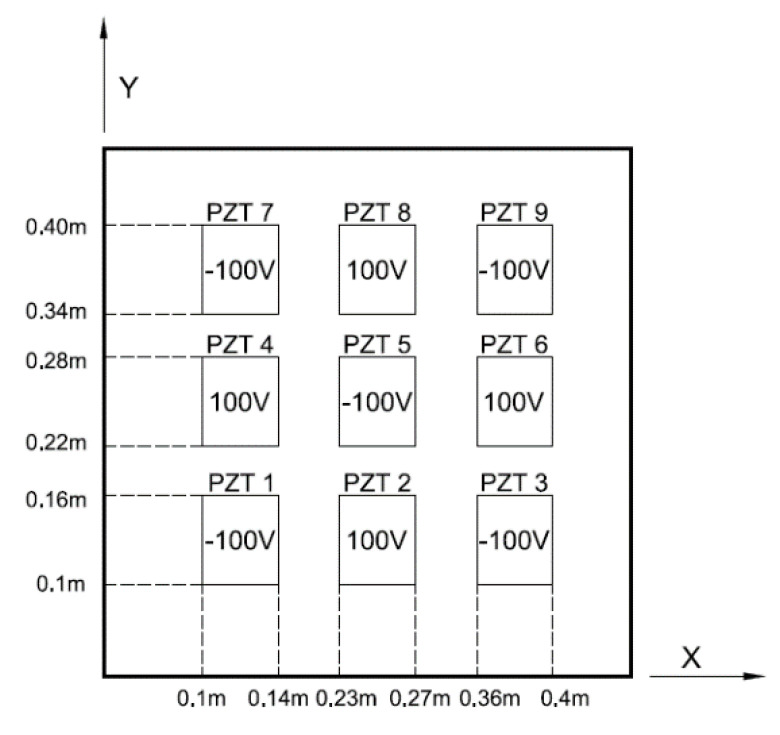
Distributed PZT actuators arranged in a 3 × 3 array surface bonded on a composite laminate.

In previous examples, the deformation of the composite laminate plate was induced by single PZT actuator. We extended present approach to distributed PZT actuators. A series of PZT actuators arranged in a 3 × 3 array was surface bonded on a composite laminate plate, as shown in Figure 15. The dimensions of PZT actuators were 40 mm × 60 mm × 0.5 mm. Electric voltages applied on the distributed PZT actuators are also shown in Figure 15. The principle of superposition was employed to determine the deformation of the composite laminate plate induced by distributed PZT actuators as follow.

(35) $$W\left(x,y\right)=\overset{9}{\underset{i=1}{\sum }}W_{i}\left(x,y\right)\;W_{i}\left(x,y\right)=\overset{\infty }{\underset{n=1}{\sum }}\overset{\infty }{\underset{m=1}{\sum }}{\left(W_{\mathrm{mn}}\right)}_{i}\sin\frac{m\pi x}{a}\sin\frac{n\pi y}{b}$$

where $W_{i}$ is the deflection of the composite laminate plate induced by the ith PZT actuator.

**Figure 16:**
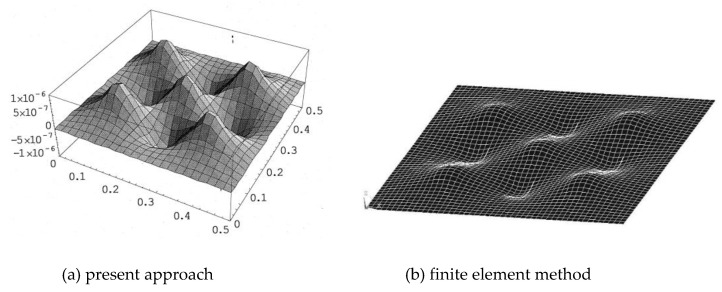
Deformed shape of the composite laminate plate induced by distributed PZT actuators.

**Figure 17:**
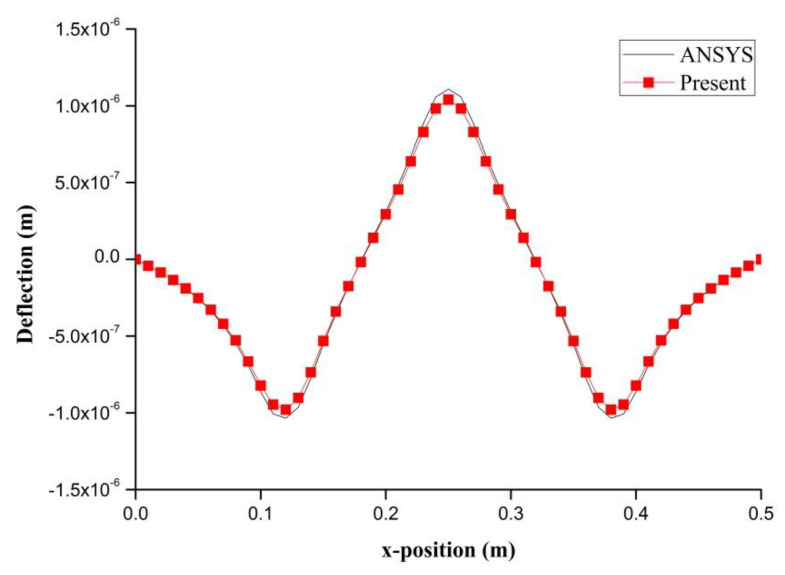
Deflection along the horizontal line (*y* = 0.25 m) of the composite laminate plate induced by distributed PZT actuators.

**Figure 18:**
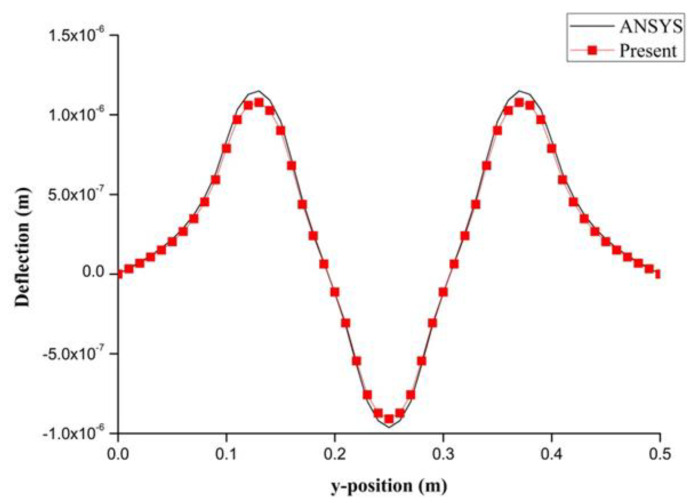
Deflection along the vertical line (*x* = 0.13 m) of the composite laminate plate induced by distributed PZT actuators.

The deformed shape of the composite laminate plate induced by distributed PZT actuators using present approach and finite element method are shown in Figure 16. The deflections of the composite laminate plate along the horizontal line (*y* = 0.25 m) and vertical line (*x* = 0.13 m) induced by the distributed PZT actuators are plotted in Figure 17 and Figure 18, respectively. It illustrates that the deformed shape of the composite laminate plate can be effectively activated by distributed PZT actuators. Good agreement is achieved between the present approach and finite element method.

## 5. Conclusions

In this work, the deformation of a composite laminate plate induced by PZT actuators was investigated. PZT actuators can be surface bonded and embedded in a composite laminate. A theoretical prediction of the deflection of a simply supported composite laminate plate induced by PZT actuators was derived using classical plate theory and composite mechanics. The feasibility of present approach was verified by finite element method. The effects of size, location, and embedded depth of PZT actuators on the deformation of composite laminate plate were investigated through a series of parametric studies. An example of distributed PZT actuators bonded on a composite laminate plate is presented to illustrate the capability of active shape control. It demonstrates that PZT actuators can be used to activate deformation of a composite laminate plate and achieve high precision shape control by distributing actuators at appropriate positions.

## Figures and Tables

**Table 1:** Material properties of Boron/Al [27].

Longitudinal Modulus *E*_1_	Transverse Modulus *E*_2_	Shear Modulus *G*_12_	Shear Modulus *G*_23_	Poisson’s Ratio *v*_12_	Poisson’s Ratio *v*_23_
227 GPa	139 GPa	57.6 GPa	49.1 GPa	0.24	0.36
